# Experimental Identification of Small Non-Coding RNAs in the Model Marine Bacterium *Ruegeria pomeroyi* DSS-3

**DOI:** 10.3389/fmicb.2016.00380

**Published:** 2016-03-29

**Authors:** Adam R. Rivers, Andrew S. Burns, Leong-Keat Chan, Mary Ann Moran

**Affiliations:** ^1^United States Department of Energy, Joint Genome InstituteWalnut Creek, CA, USA; ^2^Department of Marine Sciences, University of GeorgiaAthens, GA, USA; ^3^WaferGen Bio-Systems Inc.Fremont, CA, USA

**Keywords:** small RNA, *Ruegeria*, Roseobacter, ncRNA, sRNA

## Abstract

In oligotrophic ocean waters where bacteria are often subjected to chronic nutrient limitation, community transcriptome sequencing has pointed to the presence of highly abundant small RNAs (sRNAs). The role of sRNAs in regulating response to nutrient stress was investigated in a model heterotrophic marine bacterium *Ruegeria pomeroyi* grown in continuous culture under carbon (C) and nitrogen (N) limitation. RNAseq analysis identified 99 putative sRNAs. Sixty-nine were *cis*-encoded and located antisense to a presumed target gene. Thirty were *trans*-encoded and initial target prediction was performed computationally. The most prevalent functional roles of genes anti-sense to the *cis*-sRNAs were transport, cell-cell interactions, signal transduction, and transcriptional regulation. Most sRNAs were transcribed equally under both C and N limitation, and may be involved in a general stress response. However, 14 were regulated differentially between the C and N treatments and may respond to specific nutrient limitations. A network analysis of the predicted target genes of the *R. pomeroyi cis*-sRNAs indicated that they average fewer connections than typical protein-encoding genes, and appear to be more important in peripheral or niche-defining functions encoded in the pan genome.

## Introduction

Small non-coding RNAs are common regulators of gene expression in bacteria, including those in marine environments (Shi et al., 2009; Gifford et al., 2011). Research on marine cyanobacteria has identified several key sRNAs important in the regulation of photosystem responses to light stress in *Synechococcus* (Axmann et al., 2005; Voss et al., 2009; Gierga et al., 2012), response to iron limitation in *Prochlorococcus* (Steglich et al., 2008), and managing energy requirements in *Richelia* (Hilton et al., 2014). The sRNAs of pathogenic marine *Vibrio* have also been investigated, particularly sRNAs involved in the transition to virulence (Bardill and Hammer, 2012). Less is known about the role of sRNAs in non-pathogenic heterotrophic marine bacteria and their involvement in managing chronic nutrient limitation.

Heterotrophic marine bacteria are the primary recyclers of organic matter in the ocean, making their regulation strategies during C and N limitation important facets of marine element cycles. They must respond quickly to heterogeneity in C and nutrient availability on the microscale (resulting from patchy distributions of phytoplankton cells and nutrient plumes) and macroscale (resulting from terrestrial inputs, upwelling events, and phytoplankton blooms) (Azam and Malfatti, 2007; Stocker, 2012). For the model marine heterotroph *Ruegeria pomeroyi* DSS-3, previous studies indicate that the bacterium scavenges for alternate sources of organic C and reworks the ratios of major biomolecule classes when C limited, and exerts tight control over N uptake and export when N limited. Resource-driven changes in C:N ratios of up to 2.5-fold and in C:P ratios of up to 6-fold have been measured in *R. pomeroyi* biomass (Chan et al., 2012).

Several sRNAs are already known to be involved in bacterial regulation under C limitation. One of the first bacterial sRNAs discovered was Spot 42 in *Escherichia coli* (Sahagan and Dahlberg, 1979), which regulates expression of the galactose operon during growth on glucose (Møller et al., 2002). The sRNA SgrS controls accumulation of sugar in *E. coli* by down-regulating transport when levels of glucose-6-phosphate increase in the cell (Vanderpool and Gottesman, 2004). Mannitol transport is regulated by an sRNA in *Vibrio cholerae* (Mustachio et al., 2012).

Small RNAs involved in nitrogen metabolism have also been identified. sRNA NsiR4, discovered in the freshwater cyanobacterium *Synechocystis* sp. PCC 6803, regulates the expression of glutamine synthetase across a range of cyanobacteria (Klähn et al., 2015). In certain Gammaproteobacteria, sRNA GvcB regulates the uptake of peptides by ABC transporters (Urbanowski et al., 2000). sRNA NrsZ is induced under nitrogen limitation and helps induce swarming motility and rhamnolipid production in *Pseudomonas aeruginosa* PAO1 (Wenner et al., 2014).

To better understand the role of sRNAs in cellular regulation of C and N limitation, we sequenced transcripts from *Ruegeria pomeroyi* DSS-3 during growth in continuous culture and identified expressed sRNAs. The design allowed us to discriminate between general stress sRNAs (produced under both C and N limitation) and sRNAs specific to either C or N limitation. A study of *R. pomeroyi* sRNAs during growth on organic sulfur compounds (Burns, unpublished data) allowed us to also identify sRNAs that may be constitutively expressed. To further understand how this heterotrophic marine bacterium uses sRNA-based regulation, network analysis methods determined whether sRNAs were engaged primarily in the regulation of central metabolic processes or whether they played more important roles in peripheral or niche-defining processes.

## Methods

### Culturing

*R. pomeroyi* DSS-3 cells used for transcriptome sequencing and RT-qPCR analysis were grown in 200 ml C- and N-limited chemostats at a dilution rate of 0.042 h^−1^. Continuous culturing was used in this study in order to evaluate sRNA transcription under chronic nutrient limitation rather than the physiologically distinct process of nutrient starvation and shift to stationary phase. A basal medium with a salinity of 25 was amended with vitamins and trace metals (Table S1) and modified to establish C limitation (1 mmol l^−1^ glucose and 2.8 mmol l^−1^ NH_4_Cl) or N limitation (4.5 mmol l^−1^ glucose and 0.26 mmol l^−1^ NH_4_Cl), with three replicates run in each condition. The appropriate concentrations of limiting nutrients to produce similar biomass were determined in initial experiments. Cells were inoculated at an OD600 of 0.05 (~7.3 × 10^6^ cells ml^−1^) and cultured initially with the outflow pump turned off. After ~16 h, the flow carrying the feed medium was started. Cell cultures were mixed by constant stirring and temperature was maintained at 30°C using a circulating water bath. Air was bubbled into the culture at a flow rate of 2 ml min^−1^. At steady state, cells maintained an OD600 of 0.3. Additional details of the chemostat design are found in Chan et al. (2012). Exponential and stationary phase cultures of *R. pomeroyi* grown in ½ YTSS medium (González and Moran, 1997) were also obtained and used to confirm sRNA sizes by Northern blotting (see below).

### Transcriptomics

Samples of steady-state *R. pomeroyi* DSS-3 cells (45 ml; ~2 × 10^9^ cells) were collected from chemostats after five volume exchanges. An RNA stabilization solution (95% ethanol 5% phenol) was added to constitute 10% of the total volume and cells were pelleted by centrifugation at 4500 × g. Pellets were stored frozen at −80°C until processing. For RNA extraction, pellets were thawed and extracted using TriReagent (Molecular Research Center, Cincinnati, OH, USA). DNA was removed by the TURBO DNA-free kit (Applied Biosystems/Ambion, Austin, TX). Purified RNA was depleted of rRNA with the MicrobeExpress Kit (Ambion/Applied Biosystems, Austin, TX) and the mRNA-enriched RNA was subsequently amplified using a strand-specific protocol (MessageAmpII-Bacteria Kit; Ambion/Applied Biosystems). Using the SOLiD Whole Transcriptome Analysis Kit (Applied Biosystems), 5 μg of amplified mRNA from six samples (triplicates from both the C- and N-limitation treatments) were fragmented with RNaseIII and purified and concentrated with the RiboMinus kit (Invitogen). mRNA was examined for fragment length (Agilent 2100 Bioanalyzer) to ensure that the majority were in the 100–200 nt range. All procedures for adaptor ligation and cDNA synthesis were conducted according to the SOLiD protocol. Resultant cDNA was purified and concentrated using the MinElute PCR Purification Kit (Invitrogen), heat-denatured at 95°C, run on a Novex 6% TBE-Urea Gel (Invitrogen) under denaturing conditions with a 50 bp DNA ladder, and stained with SYBR Gold nucleic acid stain. Gel bands containing 100–200 nt cDNA (insert size 50–150 nt) were used for PCR amplification of cDNA using AmpliTaq DNA Polymerase. PCR was carried out with a 5′ SOLiD primer and a barcoded 3′ primer (using a unique barcode for each sample) for 16 cycles. Amplified cDNA was purified and concentrated using PureLink PCR Micro Kit (Invitrogen). Samples were sent to University of Washington for sequencing using a SOLiD system.

**Figure 1:**
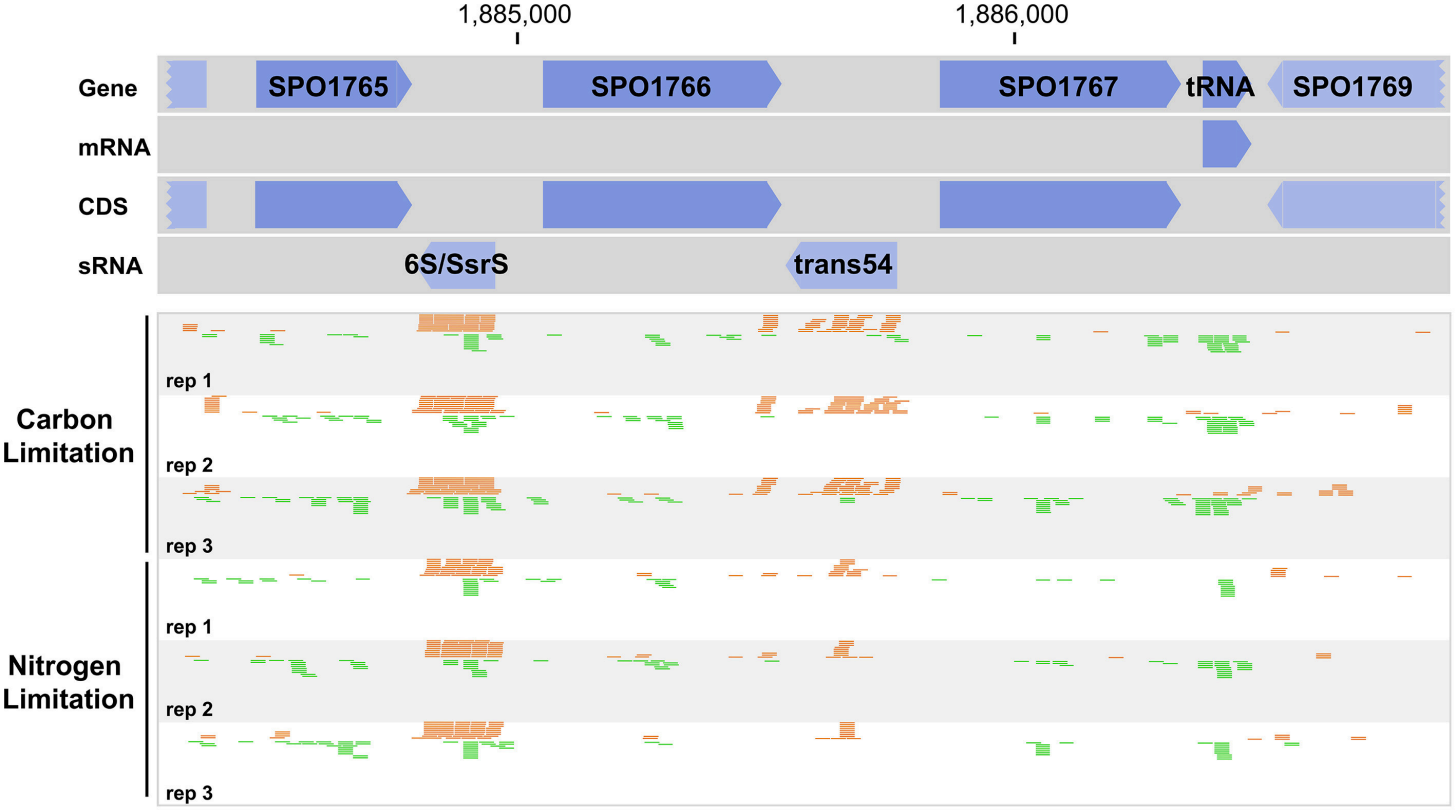
**Read mapping pattern indicative of sRNAs encoded in intergenic regions of the ***Ruegeria pomeroyi*** genome**. Orange and green reads indicate mapping on the positive and negative strand, respectively. Reads are truncated for the 6S/SsrS region, which had very high coverage.

Sequence data were mapped to the genome of *R. pomeroyi* DSS-3 [accession numbers CP000031.2 (chromosome) and CP000032.1 (megaplasmid)] using Bowtie version 0.12.9 (Langmead et al., 2009). Mapping was done in colorspace format to increase efficiency, allowing two mismatches per sequence and a 3′ trimming value of 17. BAM format files from Bowtie were analyzed in SeqMonk (http://www.bioinformatics.bbsrc.ac.uk/projects/seqmonk/). Putative sRNAs were identified by manually searching for RNA reads in intergenic regions or antisense to genes (Figure 1). Regions resembling 5′ untranslated regions were omitted. DESeq2 version 1.4.5 was used to analyze putative sRNAs for differential regulation under C and N limitation. Gene count data from both putative sRNAs and mRNAs were analyzed together since the normalization method (trimmed mean of means) assumed that a fraction of the genes did not change in abundance. Comparisons were made using an exact negative binomial test. For *cis*-sRNAs, the regulatory target was predicted to be the gene on the antisense strand. Not all *cis*-sRNAs bind and regulate their antisense transcript efficiently (Georg and Hess, 2011) but this prediction represents the most likely target if an interaction is present. For *trans*-sRNAs, the target was predicted using TargetRNA2 (Kery et al., 2014). The raw reads, BAM mapping files, and count matrix data have been deposited in EBI's ArrayExpress under accession number E-MTAB-4468.

### Northern blotting

DNA probes to central regions of abundant sRNAs were designed using Primer 3 (Untergasser et al., 2012; Table S2). The probes were labeled with biotin by modifying a procedure from Pierce Biotechnology (Rockford, IL). Hydrazide biotin was dissolved to a concentration of 50 mM in dimethyl sulfoxide (DMSO) and then diluted 1:10 in 0.1 M imadizole (pH 6). Between 7.5 and 15 nmol of oligonucleotide and 6.5 μmol of 1-ethyl-3-[3-dimethylaminopropyl]carbodiimide hydrochloride (EDC) were dissolved in 10 μl of phosphate-buffered saline. Twenty-five μl of the hydrazide biotin solution was added and the reaction was incubated at 50°C for 2 h. Labeled probe was purified by ethanol precipitation. Biotinylated RNA markers suitable for bacterial sRNA work were not commercially available, so a ladder was synthesized from the RNA Century Plus Marker Template (Life Technologies, Carlsbad, CA) using a T7 High Yield RNA Synthesis Kit (New England Biolabs, Ipswich, MA) with biotin-11-dCTP. Products were purified by 3 rounds of ethanol precipitation. The ladder is now commercially available from KeraFast (Catalog # EGA701; Boston, MA).

For each exponential and stationary phase sample, 30 μg of total RNA was separated on a 7 M urea 6% polyacrylamide gel. The gel was electro-blotted onto a nylon membrane, and RNA was crosslinked to the membrane by UV light. Probes were denatured, then hybridized overnight in ULTRAHyb-Oligo hybridization buffer (Ambion, Austin, TX). Northern blotting was carried out using the Chemiluminescent Nucleic Acid Detection Kit (Pierce Biotechnology, Rockford, IL). The size of sRNAs were estimated by measuring the migration of standard and sample bands in ImageJ (Schneider et al., 2012) and performing a regression of the standards using a Bayesian generalized linear model (gamma family, inverse link function) in the R package “arm” (Gelman and Hill, 2007).

### Reverse transcription quantitative PCR

Reverse transcription quantitative PCR (RT-qPCR) was carried out using chemostat RNA for sRNAs that were detected by Northern blotting and/or were significantly differentially expressed in the transcriptome experiments (Table 1). Primers were designed for sixteen sRNA genes plus the control genes *rpoC* and *gyrA* (Table S3). Two technical replicates were run for each of the 3 biological replicates for C- and N-limited chemostats. Amplification efficiencies were calculated using a dilution series (*n* = 8) of purified genes amplified by PCR. Data were analyzed using the R package MCMC.pqcr (Matz et al., 2013) in “classic” normalization mode in which control genes were used to account for any systematic sample variation.

**Table 1:** *****Cis*** and known regulatory sRNAs identified during growth of ***Ruegeria pomeroyi*** under C- and N-limited conditions**.

**ID**	**Size (nt)**	**Target gene locus tag**	**Target gene annotation**	**Functional category**	**Detection method**	**Fold-difference**	**FDR *p*-value**	**RT-qPCR validation**
cis1	197	SPO0003	*parA*, chromosome partitioning protein	Cell cycle control	Antisense	1.18	0.812	
cis2	153	SPO0040	ABC transporter, permease	Transport	Antisense	1.91	0.276	Yes
cis3	151	SPO0098	Peptide/opine/nickel transporter, ATP-binding	Transport	Antisense	1.57	NA	
cis4	131	SPO0108	Truncated transposase		Antisense	1.19	0.829	
cis5	277	SPO0116	Acetate kinase	Energy production	Antisense	1.99	0.150	
cis7¶	162	SPO0132	Sensor His kinase/response regulator	Signal transduction	Antisense	1.04	0.967	
cis8	127	SPO0184	Hypothetical protein		Antisense	2.70	0.066	Yes
cis9	134	SPO0185	Hypothetical protein		Antisense	2.30	NA	
cis10	280	SPO0192	*flgE*, flagellar hook protein	Motility	Antisense	1.63	NA	
**cis12**^¶^	**305**	**SPO0227**	***paxA*****, putative**	**Cell-cell interaction**	**Antisense**	**2.17**	**0.035**	**No**
cis13	155	SPO0328	Hypothetical protein		Antisense	1.67	NA	
cis14	135	SPO0411	Hypothetical protein		Antisense	1.07	NA	
cis16	139	SPO0560	Oligopeptide ABC transporter, periplasmic	Transport	Antisense	−1.31	NA	
cis17	236	SPO0574	ABC transporter, ATP binding	Transport	Antisense	−1.37	NA	
cis18	197	SPO0588	Transcriptional regulator, LysR family	Transcriptional regulation	Antisense	1.54	NA	
cis19	128	SPO0603	Hypothetical protein		Antisense	1.02	0.985	
**cis20**	**368**	**SPO0624**	**Hypothetical protein**		**Antisense**	−**2.26**	**0.001**	
cis22^¶^	218^§^	SPO0628a	Hypothetical protein		Antisense	1.12	0.788	Yes
cis23	168	SPO0649	Sugar ABC transporter, permease	Transport	Antisense	−1.42	0.485	
cis25	175	SPO0660	*naaA*, N-acetyltaurine transporter, periplasmic	Transport	Antisense	1.42	NA	
cis26	158	SPO0680	Glyoxylase family protein	Resistance	Antisense	1.16	NA	
**cis27**	**327**	**SPO0688**	**Adenylate/guanylate cyclase**	**Signal transduction**	**Antisense**	−**2.65**	**0.052**	
cis29	173	SPO0708	*ibeA*, invasion protein	Cell-cell interaction	Antisense	1.31	NA	
cis31^¶^	332	SPO0745	Hypothetical protein		Antisense	1.93	0.088	
cis32	297	SPO0749	Hypothetical protein		Antisense	1.31	0.789	
cis34	136	SPO0825	Branched-chain amino acid transport, periplasmic	Transport	Antisense	1.48	NA	
cis35^¶^	316	SPO0882	Hypothetical protein		Antisense	1.29	0.657	
cis36	187	SPO0900	Sulfate adenyltransferase	Ion metabolism	Antisense	−1.33	0.642	
cis37	188	SPO0946	Phosphomannomutase/glucomutase	Cell-cell interaction	Antisense	−1.13	NA	
cis39	161	SPO1039	Hypothetical protein		Antisense	1.10	NA	
cis40^¶^	251	SPO1059	Serine/threonine protein kinase	Signal transduction	Antisense	1.07	0.919	
cis41	255	SPO1176	Serine/threonine protein phosphatase		Antisense	−1.47	0.608	
**cis43**^¶^	**464**	**SPO1221**	**Hypothetical protein**		**Antisense**	**1.62**	**0.034**	**Yes**
cis47^¶^	164	SPO1406	Hypothetical protein		Antisense	1.34	0.457	
cis50^¶^	258	SPO1572	Serine hydroxymethyltranferase	Amino acid metabolism	Antisense	−1.40	0.549	
cis51	211	SPO1633	Hypothetical protein		Antisense	1.64	0.084	
cis52	179	SPO1658	Oligo/dipeptide ABC transporter, permease	Transport	Antisense	1.73	NA	Yes
cis55^¶^	158	SPO1929	Peptidoglycan binding protein	Cell-cell interaction	Antisense	−1.19	0.813	
cis57^¶^	260	SPO2024	Aminotransferase		Antisense	1.72	0.183	
**cis60**	**353**	**SPO2213a**	**Hypothetical protein**		**Antisense**	**2.50**	**0.001**	
cis61	201	SPO2297	Hypothetical protein		Antisense	1.34	0.604	
**cis64**	**341**	**SPO2401**	**T1SS target repeat protein**	**Cell-cell interaction**	**Antisense**	**3.05**	**0.000**	**Yes**
**cis67**	**558**	**SPO2734**	**Type I restriction/modification system**	**Cell-cell interaction**	**Antisense**	−**3.90**	**0.000**	**Yes**
cis68^¶^	413	SPO2736	Hypothetical protein		Antisense	−1.35	0.485	
cis70^¶^	224	SPO2940	Serine hydroxymethyltransferase	Amino acid metabolism	Antisense	−1.40	NA	
cis71	186	SPO2965	Ribosomal protein L33	Translation	Antisense	2.50	0.091	
cis72	455	SPO2994	Peptide/nickel/opine transporter, periplasmic	Transport	Antisense	1.62	0.479	
cis73^¶^	401	SPO2022	Valyl-tRNA synthetase	Translation	Antisense	1.22	0.805	
cis74	173	SPO3036	Metallo-B-lactamase family	Cell-cell interaction	Antisense	1.62	NA	
cis75^¶^	251	SPO3039	Polar AA ABC transporter, periplasmic	Transport	Antisense	1.13	0.865	
cis76^¶^	178	SPO3097	3-hydroxyisobutyrate dehydrogenase	Lipid metabolism	Antisense	1.45	0.510	
cis77	163	SPO3130	*xerC*, tyrosine recombinase	Recombination and repair	Antisense	1.19	0.806	
cis78^¶^	217	SPO3398	Homocysteine S-methyltransferase	Amino acid metabolism	Antisense	1.23	NA	
cis80^¶^	215	SPO3455	Adenylate/guanylate cyclase	Signal transduction	Antisense	1.33	0.585	
cis84	129	SPO3628	GNAT family acetyltransferase		Antisense	1.49	0.393	
cis86	135	SPO3666	Oxidoreductase FAD-binding		Antisense	1.09	NA	
**cis88**	**562**	**SPO3673a**	**Hypothetical protein**		**Antisense**	**2.36**	**0.000**	
cis89^¶^	326^§^	SPO3689	Transcriptional regulator, MarR family	Transcriptional regulation	Antisense	1.44	0.195	Yes
cis90^¶^	230^§^	SPO3787	sugar ABC transporter, periplasmic	Transport	Antisense	1.30	0.493	Yes
cis92	148	SPOA0008	Hypothetical protein		Antisense	1.86	NA	
cis93^¶^	221	SPOA0011	S-adenosylmethionine synthase	Coenzyme metabolism	Antisense	−1.07	NA	
**cis94**	**297**	**SPOA0026**	**3,4-dihydroxyphenylacetate 2,3-dioxygenase**		**Antisense**	−**2.75**	**0.000**	
cis95^¶^	180	SPOA0082	Hypothetical protein		Antisense	1.21	0.749	
cis97	245	SPOA0121	Sulfatase family protein		Antisense	−1.06	0.944	
cis99	404	SPOA0269	Hypothetical protein		Antisense	2.26	NA	
**cis101**	**242**	**SPOA0337**	**Hypothetical protein**		**Antisense**	**2.99**	**0.009**	
cis102	143	SPOA0342	Hypothetical protein		Antisense	−1.34	0.637	
cis103	207	SPOA0347	Hypothetical protein		Antisense	−1.38	0.604	
cis104	203	SPOA0433	Putative esterase		Antisense	1.49	NA	
riboswitch82	188	SPO1974	LuxR family autoinducer-binding regulator	Transcription	Antisense	−1.06	0.986	
4.5S RNP^¶^	177^§^	SPO1399		Translation	Rfam	NA	NA	Yes
**6S RNA**^¶^	**158**^§^				**Rfam**	**1.80**	**0.009**	**Yes**

*Fold-difference >0 indicates higher levels during C limitation and < 0 indicates higher levels during N limitation. Bold values indicate a significant difference between C and N limitation. NA = statistical testing was omitted on low-abundance sRNAs. sRNAs found previously in R. pomeroyi transcriptomes are indicated by ¶ (Burns, unpublished data). sRNAs for which Northern Blotting was carried out to confirm size predicted by transcriptome analysis are indicated by §. See Table S1 for Northern Blotting size data, genome coordinates, and COG and KEGG annotations of target genes*.

### Network analysis

A metabolic network of *R. pomeroyi* was downloaded in BioPax format from BioCyc version 19 using Pathway Tools (Caspi et al., 2014). The data were imported as a directed network into Cytoscape version 3.2.1 using the SIF import filter (Smoot et al., 2011). Proteins linked by sequential catalysis were selected and the attributes of proteins predicted to be regulated by sRNAs were analyzed relative to all protein nodes in the network. Exponential-family random graph model (ERGM) analysis was done with the statnet version 2015.11.0 package (Handcock et al., 2008) in R and the effect of nodetype on the number of edges was modeled by a Markov chain process.

## Results

### sRNA identified in *R. pomeroyi* DSS-3

**Figure 2:**
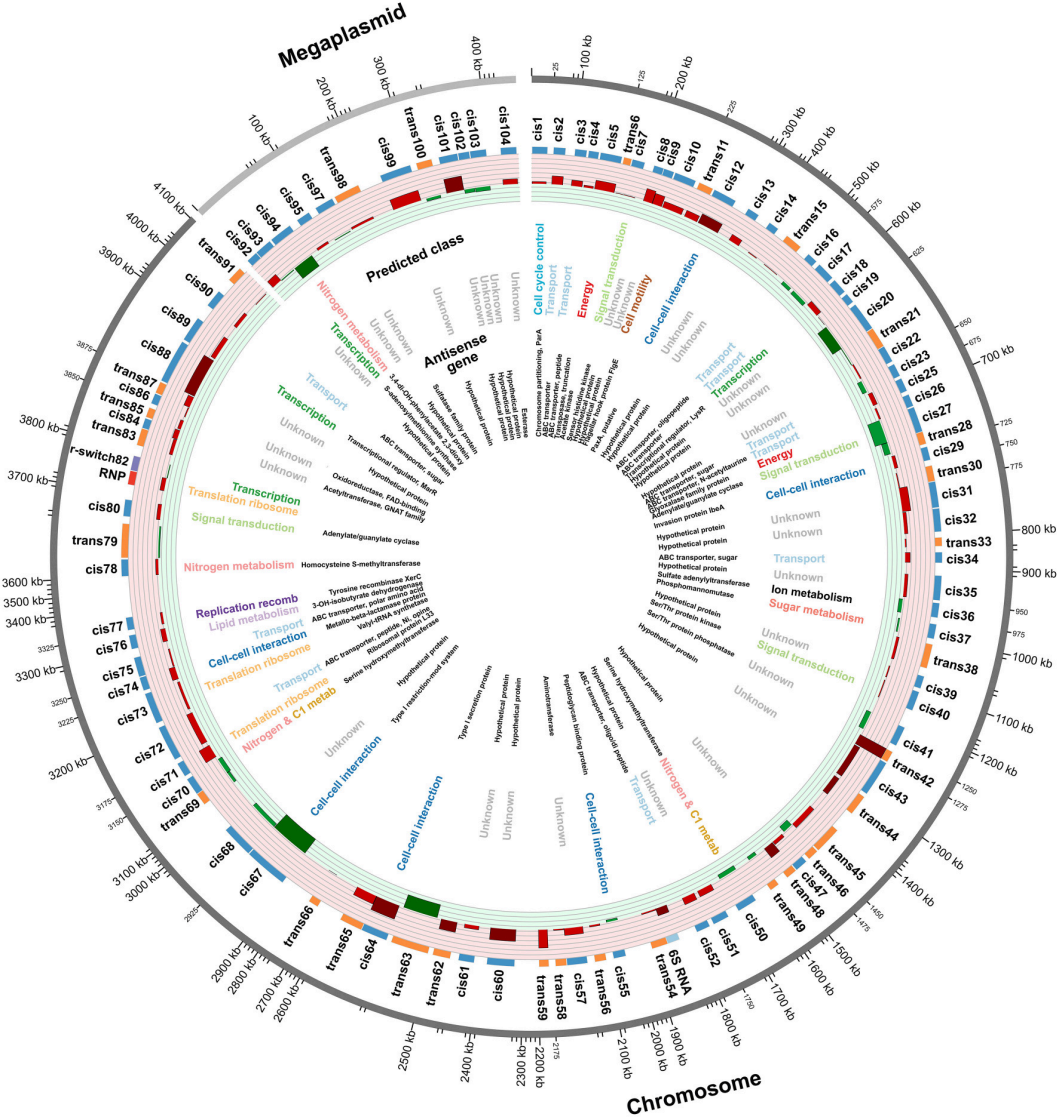
**Summary of sRNAs identified in the ***R. pomeroyi*** transcriptome during C- and N-limited growth**. Outer-to-inner rings: position in the *R. pomeroyi* chromosome or megaplasmid; sRNA ID; sRNA relative size and location, color-coded according to *cis* (blue) or *trans* (orange) mechanisms; sRNA expression level, color coded as higher during C-limited growth (red), significantly higher during C-limited growth (dark red), higher during N-limited growth (green) or significantly higher during N-limited growth (dark green), with each ring representing increments of 0.5 log_2_-fold units of differential expression; functional category of genes antisense to *cis*-sRNAs; annotation of genes antisense to *cis*-sRNAs.

A total of 99 uncharacterized sRNAs were found in *R. pomeroyi* under the growth conditions tested here. Another 3 non-coding RNAs representing known regulators were also found, including a homolog to a cobalamin riboswitch, a 6S RNA which typically associates with the RNA polymerase holoenzyme complex during stationary phase, and the 4.5S or signal recognition particle RNA which directs proteins to the cytoplasmic membrane (Table 1). sRNAs are defined by their position in the genome relative to their target genes, with *cis*-encoded sRNAs located antisense to their target and *trans*-encoded sRNAs spatially distant from their target(s) in intergenic regions of the genome. *Cis*-sRNAs often form high identity duplexes with the target transcript due to extensive complementarity, while *trans*-sRNAs form short, imperfect duplexes with limited complementarity to their mRNAs (Storz et al., 2011). The sRNAs identified in this study consisted of 69 *cis*-sRNAs and 30 *trans*-sRNAs (Figure 2).

Differential expression of sRNAs from C- and N-limited chemostat cultures was used to identify sRNAs potentially involved in nutrient-specific responses. A total of 14% of the sRNAs (14 out of 99) were differentially expressed between the two conditions compared with 10% of the 4252 protein coding genes in the transcriptome (Chan et al., 2012). More sRNAs were upregulated in the C limitation condition compared to the N limitation condition (10 of 14) (Table 1), and both *cis*- and *trans*-encoded sRNA were significantly regulated in similar proportions (Figure 2).

**Figure 3:**
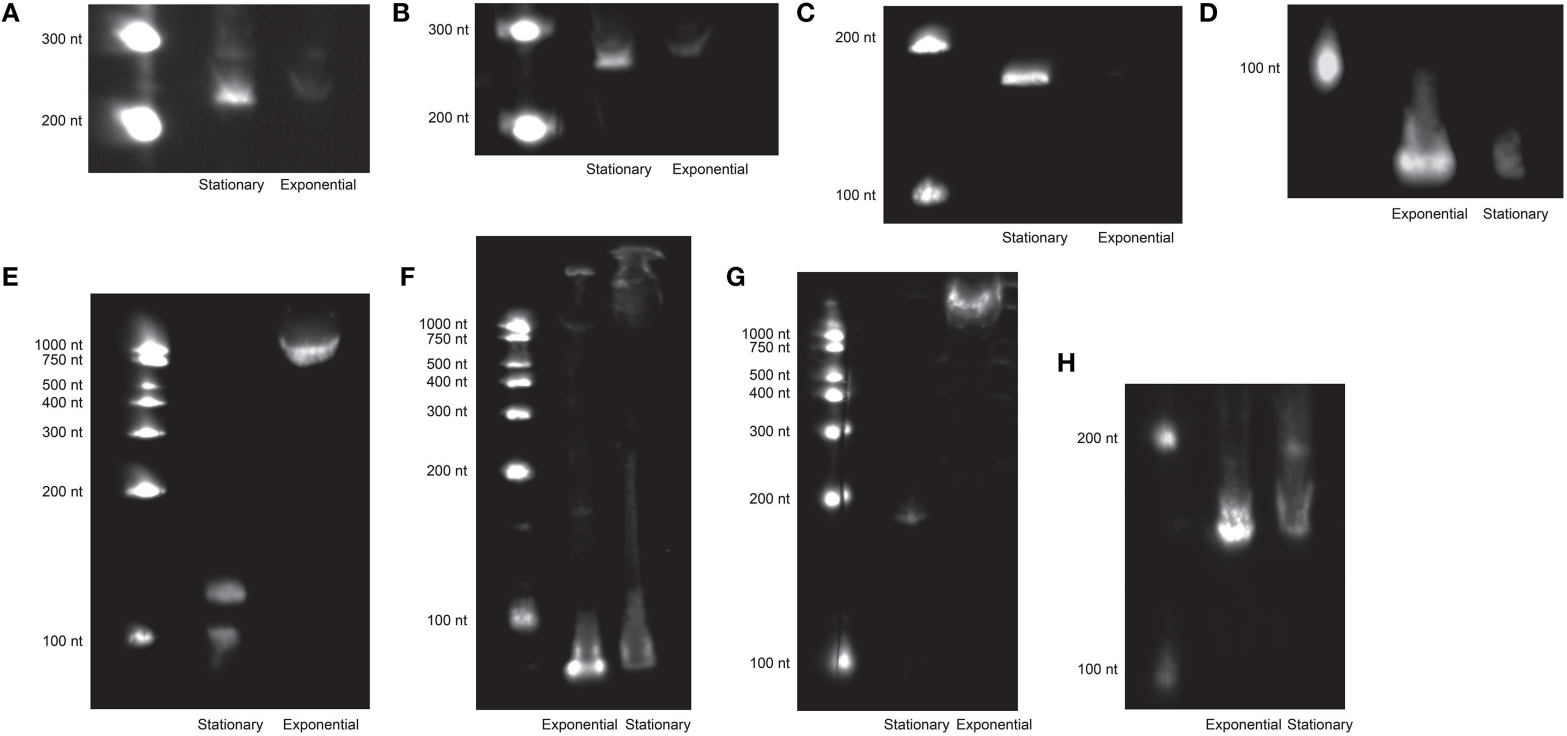
**Eight sRNAs were detected by Northern blotting (out of 11 tested). RNA on each blot is from stationary and exponential phase cultures, as indicated**. **(A)**, *cis*-22; **(B)**, *trans*-44; **(C)**, *trans*-69; **(D)**, *trans*-42; **(E)**, *trans*-62; **(F)**, 4.5S RNP; **(G)**, *cis*-89; **(H)**, 6S RNA.

To independently confirm the presence and size of sRNAs identified by transcriptome sequencing, Northern blotting was conducted for 11 abundant sRNAs. This analysis was carried out on cells grown to exponential phase (non-limiting conditions) and stationary phase (limiting conditions) because of constraints in the amount of RNA available from the chemostats. Blotting under these different conditions confirmed the presence of 8 of the sRNAs, most of which were present at higher levels in stationary phase cells compared to exponentially growing cells (Figure 3). For 4 of those, the size estimated from the transcriptome was within the 95% confidence interval of the size estimated from Northern blotting (Table S4). The 4 that fell outside the confidence intervals were all smaller than predicted from the transcriptome data, suggestive of processing of the sRNAs. To validate sRNAs with RNA obtained directly from the chemostats, reverse transcription quantitative PCR was run for sRNAs that were either significantly differentially expressed or abundant enough to be chosen for Northern blotting. Fourteen of the 16 sRNAs tested were detected; only trans42 and cis12 could not be validated by qRT-PCR (Table 1).

A previous analysis of transcription patterns of protein-encoding genes during *R. pomeroyi* growth under nutrient limiting conditions identified 190 that genes were exclusively responsive to C, N, P, or S limitation (Chan et al., 2012). Only a few of these were identified as potential targets of sRNA regulation: three genes with unknown function (SPO491, SPO1221, and SPOA0337), a response regulator (SPO3223), and *paxA* (SPO0227), whose function is discussed below.

### Functional roles

The functional category with the highest number of genes opposite the 69 *cis*-sRNAs was transport (Figure 2). All 11 transporter system proteins identified here are members of the ATP binding cassette family (ABC transporters) which is notable since *R. pomeroyi* genome also contains 39 tripartite ATP-independent periplasmic (TRAP) transporters (Moran et al., 2004). ABC transporters consume ATP when substrates are taken into the cell, while TRAP transporters rely on a sodium gradient, raising the possibility that *R. pomeroyi* more closely regulates its energetically expensive transporters. None of the sRNAs that targeted transporters had significantly different expression under C vs. N limitation. Bacterial ABC transporters typically have a periplasmic binding protein, one or two transmembrane proteins, and an ATPase, and all three protein types appeared in the target gene list for sRNA regulation.

The next largest functional category of genes antisense to *cis*-sRNAs included genes mediating cell-cell interactions, which included sRNAs predicted to regulate a gene involved in lipopolysaccharide biosynthesis (cis37) as well as the gene encoding invasion protein IbeA, shown to be involved in colonization by pathogenic *E. coli* (cis29) (Wang et al., 2011). Also in this functional category, sRNA cis12 was antisense to *paxA*, a gene encoding an RTX-like toxin that can play a role in bacterial toxicity (Kuhnert et al., 2000), while cis64 was antisense to a Type I secretion system protein that is required for export of RTX-like toxins (Linhartová et al., 2010). Other sRNAs involved in regulating protein targets potentially involved in cell-cell interactions were cis67, antisense to a Type I restriction modification gene (significantly lower under C limitation), and cis74, regulating a protein predicted to provide resistance to beta-lactam antibiotics.

Other functional categories of genes antisense to *cis*-sRNAs included nitrogen metabolism (4 sRNAs, none were differentially expressed) and gene regulation (6 sRNAs, 1 was significantly higher under N limitation). Twenty-four of the *cis*-sRNAs had hypothetical genes identified as their potential regulatory target.

Target gene prediction is more challenging for *trans*-sRNAs because they typically form imperfect and short RNA-RNA hybrids with their targets (Pain et al., 2015). Potential target genes for the *R. pomeroyi* trans-sRNAs were predicted computationally (TargetRNA2; *p* < 0.01), with the number of predicted gene targets ranging from 0 to 13 per sRNA (Table 2). Functional assignments of predicted targets were dominated by the categories of amino acid metabolism, nucleic acid metabolism, coenzyme metabolism, and transport. Functional similarity among predicted targets for a given sRNA provides a hypothesis regarding the role of trans-sRNAs in regulation. Assigned functions of predicted targets were quite diverse for most of the *R. pomeroyi* trans-sRNAs, although trans28 had several predicted target genes involved in protein catabolism, and trans58 had target genes with assigned roles in cell membrane structure (Table 2).

**Table 2:** **Predicted target genes for ***trans***-sRNAs identified during growth of ***Ruegeria pomeroyi*** under C- and N-limited conditions**.

**ID**	**Size (nt)**	**Target Gene Locus Tag**	**Target Gene Annotation**	**Functional Category**	***p*-value**	**RT-qPCR validation**
trans6	101	SPO0684	Glyoxylase family protein	Resistance	0.000	
		SPO1441	Fatty acid desaturase family protein	Lipid metabolism	0.000	
		SPO3188	Hypothetical protein		0.001	
		SPO3394	GDSL-like lipase/acylhydrolase, putative	Lipid metabolism	0.002	
		SPO2054	Cytochrome c oxidase assembly protein	Energy production	0.004	
		SPO2735	Type I restriction-modification system, R subunit	Nucleic acid metabolism	0.004	
		SPO0765	Glutamine synthetase family protein	Amino acid metabolism	0.005	
		SPO3739	Hydantoinase/oxoprolinase family protein	Amino acid metabolism	0.007	
		SPO1134	NnrU family protein		0.009	
		SPO1906	Hypothetical protein		0.009	
		SPO2498	2′-deoxycytidine 5′-triphosphate deaminase	Nucleic acid metabolism	0.009	
		SPO2912	MerR family transcriptional regulator	Transcriptional regulator	0.009	
		SPO3245	Nicotinate-nucleotide pyrophosphorylase	Coenzyme metabolism	0.009	
trans11	189	SPO0657	*naaT*, metallochaperone, putative	Coenzyme metabolism	0.003	
		SPO1108	DnaJ-like protein DjlA, putative	Post-translational modification	0.003	
		SPO3602	Hypothetical protein		0.007	
trans15	225	SPO0298	acyl-CoA dehydrogenase family protein	Lipid metabolism	0.001	
		SPO0568	2-oxoacid ferredoxin oxidoreductase	Amino acid metabolism	0.001	
		SPO2180	Hypothetical protein		0.002	
		SPO0940	Hypothetical protein		0.005	
		SPO3617	Peptidoglycan-binding protein, putative	Cell-cell interaction	0.005	
		SPO1609	Polyamine ABC transporter, ATP-binding	Transport	0.008	
		SPO0831	Xanthine dehydrogenase family, medium subunit	Nucleic acid metabolism	0.009	
		SPO0937	Hypothetical protein		0.009	
trans21	264	SPO1144	Universal stress protein family protein		0.003	
		SPO2761	Pantothenate kinase	Coenzyme metabolism	0.004	
		SPO0685	Fumarylacetoacetase	Amino acid metabolism	0.005	
		SPO2385	Benzaldehyde lyase, putative		0.007	
trans28	161	SPO0858	Methylamine utilization protein MauG, putative		0.000	
		SPO0381	Protease, putative	Protein degradation	0.001	
		SPO0129	T4 family peptidase	Protein degradation	0.004	
		SPO1697	Aminotransferase, classes I and II	Amino acid metabolism	0.006	
		SPO0934	Hypothetical protein		0.007	
		SPO0328	Hypothetical protein		0.009	
trans30	201		N/A			
trans33	111	SPO0185	Hypothetical protein		0.000	
		SPO2572	Hypothetical protein		0.001	
		SPO2073	Hypothetical protein		0.004	
		SPO0583	LysR family transcriptional regulator	Transcriptional regulator	0.005	
		SPO2679	Short chain oxidoreductase		0.006	
		SPO2900	tRNA 2-selenouridine synthase	Translation and biogenesis	0.007	
		SPO3172	Hypothetical protein		0.007	
		SPO0759	Hypothetical protein		0.008	
		SPO0872	Polysaccharide deacetylase family protein	Carbohydrate metabolism	0.009	
		SPO1845	Oxidoreductase, molybdopterin-binding		0.009	
trans38	311	SPO1267	MarR family transcriptional regulator	Transcriptional regulation	0.001	
		SPO2455	Organic solvent tolerance protein, putative	Membrane protein	0.002	
		SPO1286	Hypothetical protein		0.004	
		SPO3027	Histidinol-phosphate aminotransferase	Amino acid metabolism	0.005	
		SPO1199	Hypothetical protein		0.008	
		SPO3633	Molybdopterin converting factor, subunit 2	Coenzyme metabolism	0.008	
		SPO1311	Renal dipeptidase family protein	Protein degradation	0.009	
		SPO2536	LuxR family transcriptional regulator	Transcriptional regulation	0.009	
trans42	143^§^	SPO3876	Hypothetical protein		0.009	No
trans44	265^§^	SPO0873	Ureidoglycolate hydrolase	Nucleic acid metabolism	0.003	Yes
		SPO1532	Hypothetical protein		0.005	
		SPO0005	Hypothetical protein		0.008	
		SPO1350	Hypothetical protein		0.009	
trans45^¶^	351	SPO3050	Hypothetical protein		0.003	
		SPO2852	CzcN domain-containing protein		0.003	
		SPO2542	Biotin/lipoate binding domain-containing protein	Coenzyme metabolism	0.006	
		SPO2703	Hypothetical protein		0.008	
		SPO2977	Adenylate/guanylate cyclase	Signal transduction	0.008	
		SPO3862	Putative lipoprotein	Cell wall/membrane	0.009	
trans46	141	SPO3330	Ribonuclease R	Translation and biogenesis	0.001	
		SPO2342	Hypothetical protein		0.004	
		SPO1399	AraC family transcriptional regulator	Transcriptional regulation	0.006	
		SPO3662	Hypothetical protein		0.008	
		SPO2067	Hypothetical protein		0.010	
		SPO2790	Methylcrotonyl-CoA carboxylase, beta subunit	Lipid metabolism	0.010	
		SPO3333	Hypothetical protein		0.010	
trans48^¶^	142	SPO1762	6,7-dimethyl-8-ribityllumazine synthase	Coenzyme metabolism	0.004	
		SPO1508	Quinoprotein ethanol dehydrogenase		0.005	
		SPO3019	Xanthine dehydrogenase family, large subunit	Nucleic acid metabolism	0.006	
		SPO1029	YeeE/YedE family protein		0.007	
trans49	124	SPO1050	Phage integrase family site specific recombinase	Phage	0.001	
		SPO0323	Hypothetical protein		0.001	
		SPO0526	Acetylglutamate kinase	Amino acid metabolism	0.002	
		SPO3390	Hypothetical protein		0.003	
		SPO2630	C4-dicarboxylate transport sensor protein	Transport	0.006	
		SPO1884	Methionine synthase I	Amino acid metabolism	0.009	
		SPO3077	TldD/PmbA family protein		0.009	
		SPO1050	Phage integrase family site specific recombinase	Phage	0.001	
		SPO0323	Hypothetical protein		0.001	
trans54	207		N/A			
trans56	146	SPO0201	Hypothetical protein		0.001	
		SPO1217	DNA-binding protein, putative	Transcriptional regulation	0.001	
		SPO0547	Hypothetical protein		0.002	
		SPO1032	Hypothetical protein		0.006	
		SPO2286	Autoinducer-binding regulator LuxR	Transcriptional regulation	0.007	
		SPO0201	Hypothetical protein		0.001	
trans58	141	SPO3492	Hypothetical protein		0.000	
		SPO2182	Permease, putative	Transport	0.001	
		SPO0236	Glycerophosphoryl diester phosphodiesterase putative	Lipid metabolism	0.001	
		SPO0950	Uracil-DNA glycosylase, putative	Nucleic acid metabolism	0.001	
		SPO3756	OmpA domain-containing protein	Cell wall/membrane	0.004	
		SPO1732	Single-stranded-DNA-specific exonuclease RecJ	Recombination and repair	0.005	
		SPO0965	Acetyltransferase		0.006	
		SPO1596	Hypothetical protein		0.006	
		SPO0846	Phosphopantetheinyl transferase PptA, putative	Coenzyme metabolism	0.008	
		SPO1099	Hypothetical protein		0.010	
trans59^¶^	124		N/A			
trans62^¶^	225^§^	SPO0491	Hypothetical protein		0.001	Yes
		SPO2176	Hypothetical protein		0.003	
		SPO2943	Alpha/beta fold family hydrolase		0.004	
		SPO2635	Phosphoadenosine phosphosulfate reductase	Sulfur metabolism	0.004	
		SPO2397	Carbon monoxide dehydrogenase, large subunit		0.005	
		SPO2759	NUDIX family hydrolase	Nucleic acid metabolism	0.005	
		SPO0294	NUDIX family hydrolase	Nucleic acid metabolism	0.007	
		SPO0571	PKD domain-containing protein		0.007	
		SPO1376	Glycosyl transferase, group 2 family protein	Carbohydrate metabolism	0.008	
		SPO2218	Excinuclease ABC subunit A	Recombination and repair	0.008	
		SPO2718	Hypothetical protein		0.008	
		SPO2640	XdhC/CoxI family protein	Nucleic acid metabolism	0.009	
		SPO3493	Transporter, putative	Transport	0.009	
trans63^¶^	488	SPO0331	Thiol:disulfide interchange protein, putative		0.000	
		SPO0965	Acetyltransferase		0.002	
		SPO3587	Hypothetical protein		0.004	
		SPO1527	Hypothetical protein		0.005	
		SPO2008	Polyamine ABC transporter, permease protein	Transport	0.009	
		SPO0305	AzlC family protein	Amino acid metabolism	0.010	
		SPO0773	Acetyl-CoA acyltransferase/thiolase family	Lipid metabolism	0.010	
		SPO2147	Hypothetical protein		0.010	
trans65	295	SPO1043	Hypothetical protein		0.000	
		SPO3750	Hypothetical protein		0.001	
		SPO2911	Thioesterase family protein		0.003	
		SPO2543	GntR family transcriptional regulator	Transcriptional regulation	0.004	
		SPO2551	Peptide/opine/nickel uptake ATP-binding protein	Transport	0.008	
		SPO0919	MarR family transcriptional regulator	Transcriptional regulation	0.009	
trans66	130	SPO0164	Oxidoreductase, FMN nucleotide-disulfide		0.003	
		SPO1125	Hypothetical protein		0.006	
		SPO1226	Putative lipoprotein	Lipid metabolism	0.006	
		SPO1510	Efflux ABC transporter, permease protein	Transport	0.007	
		SPO3172	Hypothetical protein		0.009	
trans69	160^§^	SPO0934	Hypothetical protein		0.007	Yes
		SPO3650	Adenylate/guanylate cyclase	Signal transduction	0.009	
trans79	436	SPO3223	Response regulator	Transcriptional regulation	0.002	
		SPO1656	Oligopeptide/dipeptide ABC, ATP-binding	Transport	0.003	
		SPO0310	Molybdopterin biosynthesis protein MoeA	Coenzyme metabolism	0.004	
		SPO1432	Rhodanese domain-containing protein		0.006	
		SPO0078	Ribosomal subunit interface protein, putative	Translation and biogenesis	0.007	
		SPO2314	DsbE periplasmic thiol:disulfide oxidoreductase	Post-translational modification	0.007	
trans83	121	SPO1889	Alcohol dehydrogenase, zinc-containing		0.002	
		SPO0829	Hypothetical protein		0.002	
		SPO2580	Hypothetical protein		0.003	
		SPO1905	Fumarate hydratase	Energy production	0.004	
		SPO2296	Hypo#thetical protein		0.006	
		SPO0451	D-alanyl-D-alanine carboxypeptidase	Amino acid metabolism	0.007	
		SPO1189	Hypothetical protein		0.007	
		SPO1273	FAD-dependent thymidylate synthase	Nucleic acid metabolism	0.007	
		SPO2196	Diaminopropionate ammonia-lyase	Amino acid metabolism	0.007	
		SPO2407	ISSpo6, transposase orfB	Recombination and repair	0.007	
trans85^¶^	116	SPO0295	Hypothetical protein		0.003	
		SPO2757	EF hand domain-containing protein		0.007	
trans87^¶^	150	SPO1856	ribonuclease BN, putative	Translation and biogenesis	0.009	
		SPO3851	HemY domain-containing protein	Coenzyme metabolism	0.009	
trans91^¶^	201	SPO0387	Hypothetical protein		0.002	
		SPO2407	ISSpo6, transposase orfB	Recombination and repair	0.009	
trans98	337	SPO0220	rRNA large subunit methyltransferase		0.001	
		SPO3402	Amino acid transporter LysE	Transport	0.002	
		SPO1802	Hypothetical protein		0.003	
		SPO1481	Hypothetical protein		0.003	
		SPO2249	Hypothetical protein		0.005	
		SPO0879	acyl-CoA dehydrogenase family protein	Lipid metabolism	0.005	
		SPO3823	50S ribosomal protein L23	Translation and biogenesis	0.005	
		SPO3600	Pyruvate kinase	Carbohydrate metabolism	0.007	
		SPO1395	AraC family transcriptional regulator	Transcriptional regulation	0.010	
trans100	201	SPO0259	Hypothetical protein		0.000	
		SPO1198	Hypothetical protein		0.006	

*Target genes were predicted with a p < 0.01 using TargetRNA2. sRNAs found previously in R. pomeroyi transcriptomes are indicated by ¶ (Burns, unpublished data). Trans-sRNAs for which Northern blotting was carried out to confirm size predicted by transcriptome analysis are indicated by §. See Table S1 for Northern Blotting size data and genome coordinates. N/A, no significant target genes were predicted*.

A non-coding RNA with homology to the 6S RNA was also found in the *R. pomeroyi* transcriptome. In *E. coli* and many other bacteria, 6S RNA is a global regulator that downregulates transcription of multiple genes when the bacterium is under stress, including during nutrient limitation (Cavanagh and Wassarman, 2014). In *R. pomeroyi*, the 6S RNA homolog was significantly upregulated under C limitation relative to N limitation (Table 1), and it was also noted in a previous study of non-coding RNA expression in this bacterium during sulfur metabolism (Burns, unpublished data) (Table S4).

### Mode of action of sRNAs

**Figure 4:**
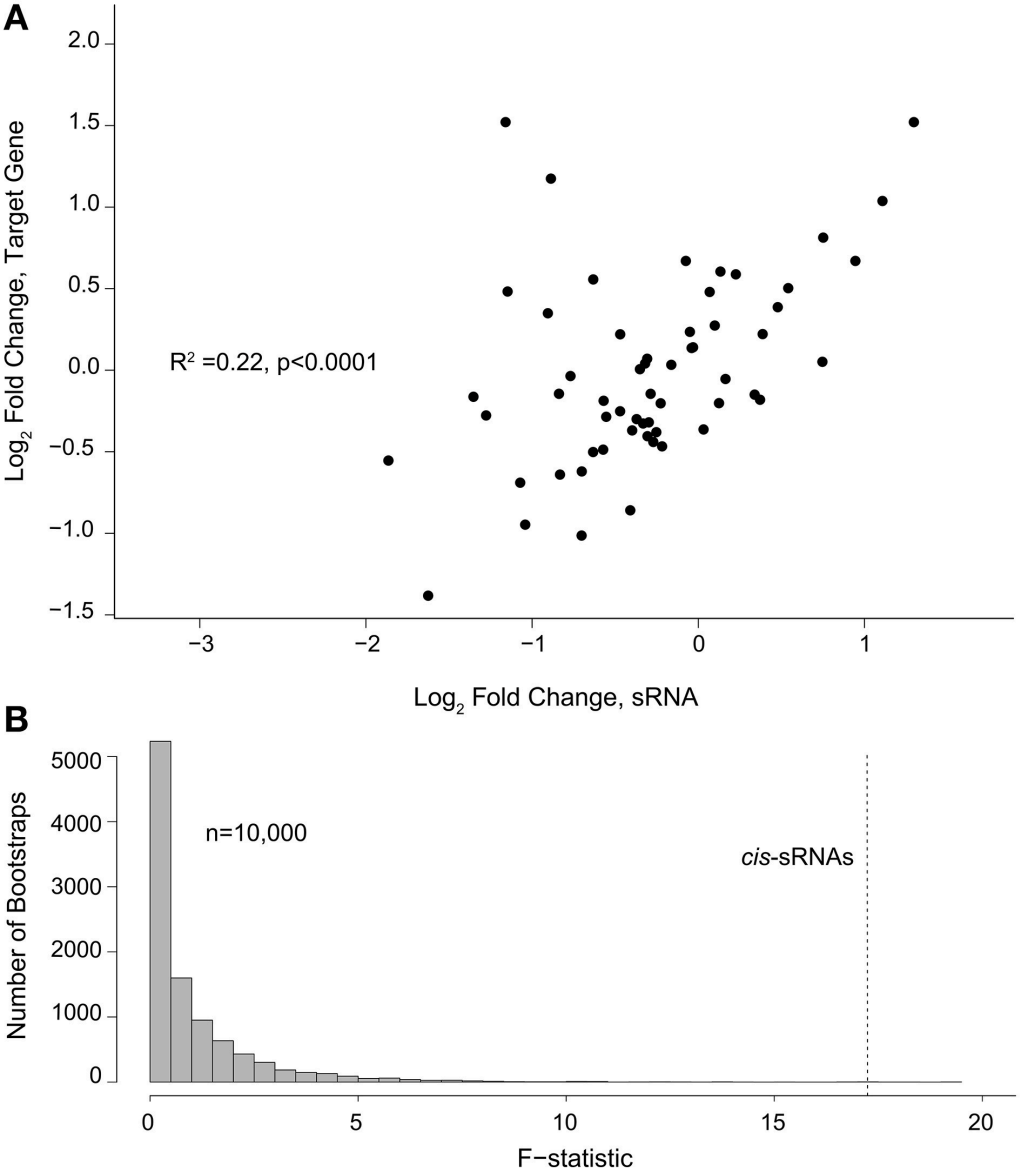
**(A)** Changes of *cis*-sRNAs and their predicted gene target (log_2_ fold-change) under C vs. N limitation. **(B)** Distribution of F-statistics for correlations from 10,000 random pairings of sRNAs and target genes (null model). The F-statistic for the correlation from **(A)** is indicated with a dashed line.

sRNAs and their regulatory targets may or may not have positively correlated patterns of expression, depending on whether the sRNAs affect transcript stability or instead work at the level of translation, and whether they act as activators or repressors. To determine whether there was any consistency in sRNA mode of action, the fold-difference between C- and N-limiting conditions for predicted target genes was plotted against the fold-difference for their corresponding *cis*-sRNAs. A weak but significant positive correlation was observed (*R*^2^ = 0.22), suggesting that the most common *cis*-sRNA mode of action under C and N limitation is as a positive regulator of mRNA levels (Figure 4A). To test the likelihood that this outcome could occur by chance, the antisense protein coding genes and sRNAs were paired randomly in 10,000 bootstrap analyses. F statistics for the actual pairs of antisense genes and *cis*-sRNAs had a value of 17.1 and was significantly higher than the F statistic of the median null sample (0.45) (Figure 4B).

**Figure 5:**
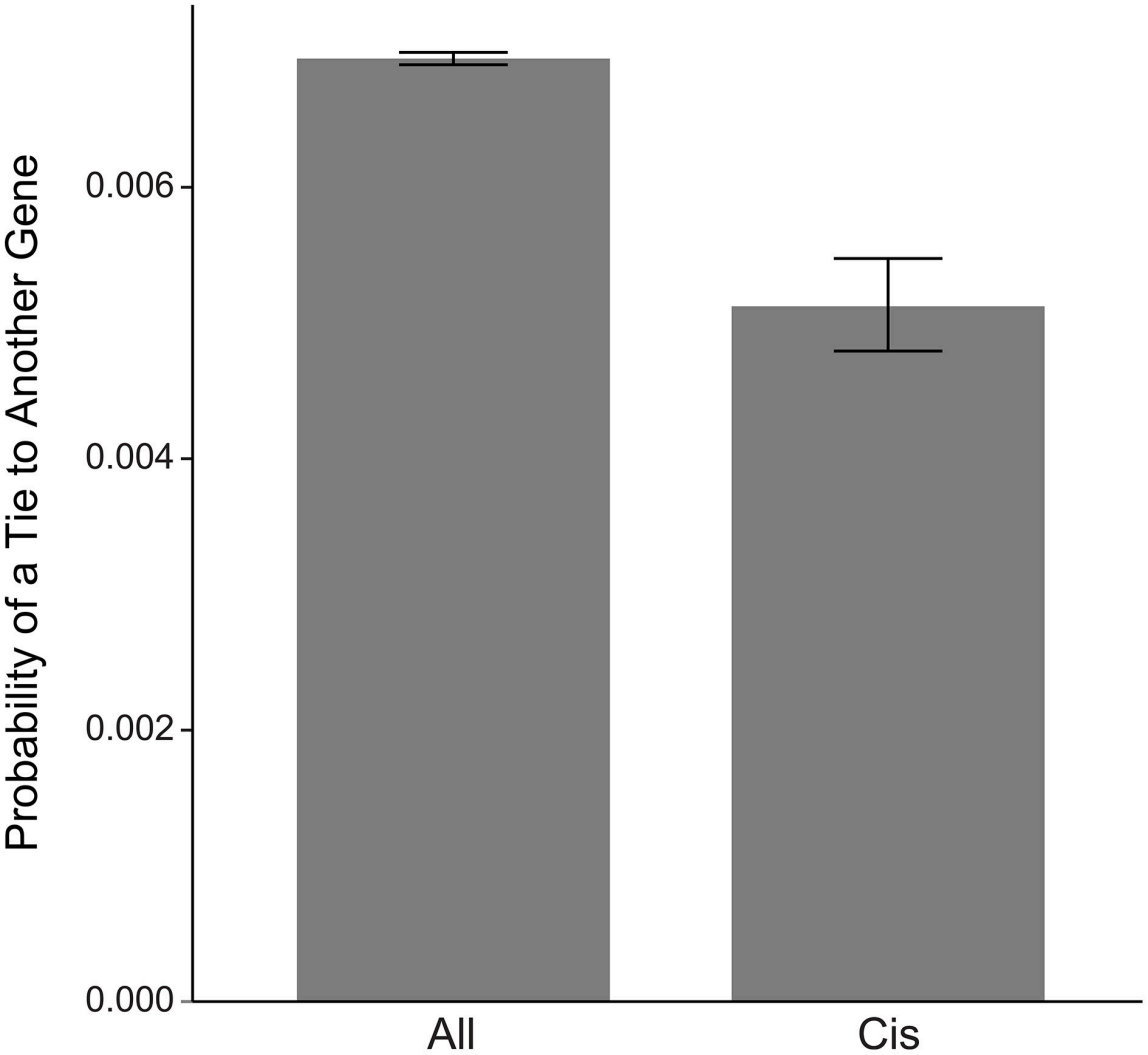
**ERGM network analysis of connectedness (probability of links to other genes) for genes regulated by ***cis***-sRNAs compared to all genes in the ***R. pomeroyi*** genome**.

We were interested in understanding whether sRNAs play more important roles in the regulation of central metabolism (typically encoded in the core genome) or the regulation of peripheral or non-core metabolic processes (encoded in the pan genome). A metabolic map based on the *R. pomeroyi* genome (BioCyc Database Collection; http://biocyc.org) was used in a network analysis of the 22 genes antisense to *cis*-RNAs (Figure 5). Exponential family random graph models (ERGM) were used to independently assess the differences in connectedness for genes antisense to *cis*-sRNAs compared to all genes. These models behave like generalized linear models in which the response variable is the structure of a network and the predictor variables are categorical or continuous node or edge attributes and emerging network statistics. The vector of response variable coefficients can then be estimated using Markov Chain Monte Carlo (MCMC) simulations and the Akaike Information Criterion (AIC) to assess model fit. Genes antisense to *cis*-sRNAs had a significantly lower probability of interacting with other genes in the network compared to the average of all genes (Figure 5).

## Discussion

### Carbon vs. nitrogen limitation

Carbon and nitrogen limitation represent major challenges to the growth of heterotrophic bacteria and affect both anabolic and catabolic processes. Of the 14 sRNAs that showed significant differential regulation in the comparison between C and N limitation, most were higher under C limitation (10 of 14) (Table 1). This may reflect a need by *R. pomeroyi* for more complex regulatory strategies for the diverse mixture of organic C molecules found in seawater compared to a more constrained suite of inorganic N species and organic N molecules (Singer et al., 2012; Medeiros et al., 2015).

Transporter genes made up the largest functional class of predicted target genes of *R. pomeroyi cis*-sRNAs. One of the 11 ABC transporter genes in this class encodes an experimentally verified transporter for the sulfonate *N*-acetyltaurine (Denger et al., 2011), a nitrogen- and sulfur-containing organic compound important in diatom-derived organic matter (Durham et al., 2015). The remainder of the transporters had only general annotations based on homology to previously characterized amino acid, peptide, and sugar transporter systems (7 target proteins), or had no substrate assigned (3 target proteins). None of these sRNAs target genes were differentially regulated under C vs. N limitation.

Two target genes that may work together in the synthesis and export of a toxin were predicted to be under the control of sRNAs (cis12 and cis24) (Table 1), with neither differentially regulated under C vs. N limitation. One of them is the *R. pomeroyi* gene annotated as *paxA*, a gene first identified in bacterial pathogen *Pasteurella aerogenes* to encode an RTX toxin (Kuhnert et al., 2000), a class of protein toxins that form pores in eukaryotic host cells (Benz, 2016). The second gene is the target repeat protein of *R. pomeroyi*'s type I secretion system (T1SS), required for the export of RTX toxins by Gram negative bacteria (Welch, 2001). PaxA has been reported to account for as much as 50% of proteins exported by *R. pomeroyi* when grown in laboratory medium enriched by the addition of yeast extract, but as little as 3% in conditions mimicking natural seawater (Christie-Oleza et al., 2015).

Although not differentially transcribed, two *cis*-sRNAs were predicted to regulate components of methionine metabolism, one encoded antisense to *metK* (S-adenosylmethionine synthase; cis93) and one encoded antisense to a homocysteine S-methyltransferase gene (cis78). Two others were predicted to regulate proteins involved in *N*-acetyltaurine use. One was transporter component *naaA* (cis25) and the other a catabolic metallochaperone gene *naaT* (a predicted target gene for trans11). Other sRNAs that were present but not differentially expressed between C and N limiting conditions included those predicted to regulate a flagellar hook protein (cis10) and a methylamine utilization gene *mauG* (a predicted target gene for trans28).

Thirty sRNAs identified here were also expressed by *R. pomeroyi* in a study of organic sulfur metabolism (Burns, unpublished data), and these represent candidates for constitutively expressed sRNAs (Table S4). The distribution of functional categories between the possible constitutively expressed sRNAs and those predicted to be involved specifically in nutrient limitation was similar.

### Regulatory mechanisms of sRNAs in *R. pomeroyi*

The regulatory mechanisms of bacterial sRNA are typically based on direct RNA-RNA binding with a target mRNA, with some exceptions for sRNAs that interact with proteins (Gottesman and Storz, 2011). They can affect gene expression in several ways, including changing mRNA half-life through stabilization or degradation, and modulation of translation through changes in mRNA secondary structure that open or occlude the ribosome binding site (Wassarman et al., 1999; Papenfort and Vogel, 2014). Each of these mechanisms predicts a different pattern when comparing the change in abundance of sRNAs and their targets. In *R. pomeroyi*, a statistically significant positive correlation with a slope of ~0.5 was found between *cis*-sRNAs and their targets (Figure 4A). A bootstrapping analysis with random pairing of predicted target coding genes and sRNAs indicated that the correlation had a very low probability of occurring due to chance or to an underlying bias in the data types. This pattern of target/sRNA expression change suggests that the most common regulatory mechanisms of *cis*-sRNAs in *R. pomeroyi* growing under C and N limitation are through stabilization of target gene transcripts or possibly transcriptional activation, although there are relatively few examples of bacterial sRNA transcriptional activators in the literature (Goodson et al., 2012). Some sRNAs fall into the upper left and lower right quadrants of Figure 4A, and these may represent negative regulatory mechanisms. The majority of modes of sRNA interactions described thus far in the literature rely on translational repression or mRNA degradation, although few studies have also looked at genome wide patterns of sRNA regulation. It should be noted that this analysis can only capture sRNAs which regulate by RNA-RNA interaction.

### Centrality of genes regulated by sRNAs

sRNAs have the potential to participate in expansion of the functional capabilities of marine bacteria by facilitating regulation of genes acquired by horizontal transfer. They are less costly to maintain than protein regulators, and their regulatory abilities are encoded directly with the gene being transferred. *Trans*-sRNAs may also play an important role in regulation of transferred genes, and among members of the Roseobacter clade, the gene encoding the Hfq protein (used by some *trans*-acting sRNAs) is one on the most conserved (Newton et al., 2010). sRNAs have also been identified in pathogenicity islands and phage genomes (Gottesman and Storz, 2011). To gain insight into the issue of which classes of genes are more likely to be targeted by sRNAs, the location of *cis*-RNA-regulated genes within the metabolic network of *R. pomeroyi* was analyzed. The ERGM network analysis revealed that genes identified as targets of *cis*-RNAs are about 20% less connected than the average gene. Genes that are part of the core genome are more often included in metabolic networks than those in the pan genome, suggesting that estimates based on metabolic networks may actually understate a central metabolism vs. peripheral function effect. Transporter genes were the largest group of sRNA targets in *R. pomeroyi*, which is consistent with this possible bias. Only 22% of sRNA target genes were present in the metabolic network while 39% of the total genes were present (*p* < 0.0, *Z*-test).

## Conclusions

The results of this study emphasize the number and variety of sRNAs produced by a heterotrophic marine bacterium and the need for additional research into the role of sRNAs in facilitating ecological adaptations. sRNAs represent an additional layer of regulation governing the cycling of C and nutrients in the ocean that affects the interpretation of transcriptome data both in model organisms and marine microbial communities.

## Author contributions

AR designed the project, conducted the research, and wrote the paper. AB designed the project, conducted the research, and wrote the paper. LC conducted the research. MM designed the project and wrote the paper.

### Conflict of interest statement

The authors declare that the research was conducted in the absence of any commercial or financial relationships that could be construed as a potential conflict of interest.
