# Supplementary material for: Experimental Identification of Small Non-Coding RNAs in the Model Marine Bacterium Ruegeria pomeroyi DSS-3
Source: Front Microbiol. 2016 Mar 29;7:380. doi: 10.3389/fmicb.2016.00380 (PMC4809877; doi:10.3389/fmicb.2016.00380)
Supplement: Supplementary file 1 [file Table1.DOCX]

Supplementary Material

Experimental identification of small non-coding RNAs in the model marine bacterium *Ruegeria pomeroyi* DSS-3

Adam R. Rivers, Andrew S. Burns, Leong-Keat Chan, Mary Ann Moran*

*** Correspondence:** mmoran@uga.edu

# Supplementary Table S1

Table S1. Chemostat culture medium. For glucose and ammonium, the concentration in the limiting medium is given in parentheses.

| Compound | | Concentration | | | |  |
| --- | --- | --- | --- | --- | --- | --- |
| *Macronutrient* | | | | |  |  |
| Glucose (C_6_H_12_O_6_) | | 4.50 mmol l^-1^ ( 1.00 mmol l^-1^) | | | |  |
| Ammonium (NH_4_Cl) | | 2.80 mmol l^-1^ ( 0.26 mmol l^-1^) | | | |  |
| Phosphate (KH_2_PO_4_) | | 0.50 mmol l^-1^ | | | |  |
|  | |  | | | |  |
| *Buffer, iron, and salt*^a^ | | | | |  |  |
| Bis-tris propane (C_11_H_26_N_2_O_6_)^b^ | | | 9.91 mmol l^-1^ |  |  |  |
| CaCl_2_•2H_2_O | | 7.42 mmol l^-1^ | | | |  |
| MgCl_2_ | | 106.21 mmol l^-1^ | | | |  |
| Iron-EDTA (C_10_H_12_FeN_2_NaO_8_)^c^ | | 67.52 μmol l^-1^ | | | |  |
| KCl | | 10.64 mmol l^-1^ | | | |  |
| NaCl | | 198.49 mmol l^-1^ | | | |  |
|  | |  | | | |  |
| *Trace element* | | | | |  |  |
| C_6_H_9_NO_6_ | 12.95 μmol l^-1^ | | | | | |
| CoCl_2_•6H_2_O | | 0.80 μmol l^-1^ | | | |  |
| Na_2_SeO_3_ | | 1.87 μmol l^-1^ | | | |  |
| Na_2_WO_4_•2H_2_O | | 0.55 μmol l^-1^ | | | |  |
| BaCl_2_•2H_2_O | | 0.49 μmol l^-1^ | | | |  |
| CuSO_4_ | | 55.57 nmol l^-1^ | | | |  |
| MnSO_4_•H_2_O | | 0.77 μmol l^-1^ | | | |  |
| ZnSO_4_•7H_2_O | | 0.54 μmol l^-1^ | | | |  |
| Na_2_MoO_4_•2H_2_O | | 0.57 μmol l^-1^ | | | |  |
| Na_2_SiO_3_•9H_2_O | | 0.45 μmol l^-1^ | | | |  |
| SrCl_2_•6H_2_O | | 0.19 mmol l^-1^ | | | |  |
| NiCl_2_•6H_2_O | | 96.32 nmol l^-1^ | | | |  |
|  | |  | | | |  |
| *Vitamin* | | | | |  |  |
| Thiamine (B1) | | 0.15 μmol l^-1^ | | | | |
| Nicotinic acid (B3) | | 0.40 μmol l^-1^ | | | |  |
| Pyridoxine-HCl (B6) | | 0.48 μmol l^-1^ | | | |  |
| Folic acid (B9) | | 44.86 nmol l^-1^ | | | |  |
| Cyanocobalamin (B12) | | 7.30 nmol l^-1^ | | | |  |
| Riboflavin (B2) | | 0.13 μmol l^-1^ | | | |  |
| Pantothenic acid (B5) | | 0.23 μmol l^-1^ | | | |  |
| Biotin (B7) | | 81.05 nmol l^-1^ | | | |  |
| *p*-aminobenzoic acid (B10) | | 0.36 μmol l^-1^ | | | |  |

^a^Final salts concentration is 23.6 g l^-1^ (salinity of ~25).

^b^Bis-tris propane, 1,3-bis(tris(hydroxymethyl)methylamino)propane.

^c^Iron-EDTA, ethylenediaminetetraacetic acid iron (III) sodium salts.
