# Supplementary material for: Experimental Identification of Small Non-Coding RNAs in the Model Marine Bacterium Ruegeria pomeroyi DSS-3
Source: Front Microbiol. 2016 Mar 29;7:380. doi: 10.3389/fmicb.2016.00380 (PMC4809877; doi:10.3389/fmicb.2016.00380)
Supplement: Supplementary file 2 [file Table2.DOCX]

Supplementary Material

Experimental identification of small non-coding RNAs in the model marine bacterium *Ruegeria pomeroyi* DSS-3

Adam R. Rivers, Andrew S. Burns, Leong-Keat Chan, Mary Ann Moran*

*** Correspondence:** mmoran@uga.edu

# Supplementary Table S2

Table S2. Probes used for Northern blotting verification of 11 *R. pomeroyi* sRNAs initially identified by RNAseq analysis.

| **Northern probe** | **Sequence** |
| --- | --- |
| DSS3_cis90 | GAGGCGGTCTATCTGTTCCTGCAATGGCTCAGCTCGACCCG |
| DSS3_trans62 | GTGCCGCCCAGGCTGAGCAGCACGACGAGCAGCAGAATGAT |
| DSS3_cis22 | CTGACCCCTCAGGCCAGACCCGGCACTGGAAGACGCCGGGA |
| DSS3_trans6 | GGCCAACAGGCTCGACCGGGGCGGGGACCCGCCAAGGACTG |
| DSS3_cis88 | CGCAAAGGTAAACGGCGAATCAAAAGCAGCCCACGACACCA |
| DSS3_trans69 | TCGGGGCGGACGCAAGCTACAAACCTGCGCCCGCTGCAAAC |
| DSS3_trans81 | AGCGAACGGCGGTGAAAGTGAGAGGCTGGACATCGACCCAA |
| DSS3_trans42 | GTACCGGACTTGCCTCCATCAATCCAAGTCATTGAATAACT |
| DSS3_cis53 | AGGTGGGCGCCAGATAATCAGGCCAGGACCTGAACAGAGCC |
| DSS3_trans43 | CTTTTGGCCAGCTTACACCGTTTTCCCAACCACAGTGAGGG |
| DSS3_trans44 | CACTCTGTTCGGGCCCAGCTGTACCCGCTTGGGGAATTGGC |
| DSS3_trans89 | GGTTCTCTCCGGCAGGGTGGCCAGGCCAGGTGTAGCTGCGC |
