# Supplementary material for: Experimental Identification of Small Non-Coding RNAs in the Model Marine Bacterium Ruegeria pomeroyi DSS-3
Source: Front Microbiol. 2016 Mar 29;7:380. doi: 10.3389/fmicb.2016.00380 (PMC4809877; doi:10.3389/fmicb.2016.00380)
Supplement: Supplementary file 3 [file Table3.DOCX]

Supplementary Material

Experimental identification of small non-coding RNAs in the model marine bacterium *Ruegeria pomeroyi* DSS-3

Adam R. Rivers, Andrew S. Burns, Leong-Keat Chan, Mary Ann Moran*

*** Correspondence:** mmoran@uga.edu

# Supplementary Table S3

Table S3. Forward (F) and reverse (R) primers used for reverse transcription quantitative PCR verification of 16 *R. pomeroyi* sRNAs initially identified by RNAseq analysis.

| **qRT-PCR Primer** | **Sequence** |  |
| --- | --- | --- |
| DSS3_cis12_58F | CTCGGGTTCGGGTTTCGG | |
| DSS3_cis12_141R | CCCGAACCCGAACCGAAG | |
| DSS3_cis22_72F | AACCACCCCCGATCAAGTTC | |
| DSS3_cis22_146R | CCGCCTGGTAAACATCCTGA | |
| DSS3_cis53_58F | CTGGCTCTGTTCAGGTCCTG | |
| DSS3_cis53_120R | TCCCGAGGACCTGTATGTACA | |
| DSS3_trans42_32F | ACCTCTCACTTGGTATCCTAAGT | |
| DSS3_trans42_107R | TTGTGATGGAGGCGAGTACC | |
| DSS3_trans44_103F | GAAAAGGCAGGCCAATTCCC | |
| DSS3_trans44_179R | TCCAGCACTTCGTCCTCAAC | |
| DSS3_trans62_19F | AGCAACGCCAGTATTACCCG | |
| DSS3_trans62_112R | ACGACGAGCAGCAGAATGAT | |
| DSS3_trans69_35F | GTCGAGTGTTGGGACGAGTT | |
| DSS3_trans69_123R | AAATCAGACGGCCGATGGTC | |
| DSS3_trans81_73F | GTCGATGTCCAGCCTCTCAC | |
| DSS3_trans81_132R | CTAGAGGGTTCCAAGTGCGG | |
| DSS3_trans89_42F | ACAGTGTTGCAATGAGCTGC | |
| DSS3_trans89_125R | GCCCCAGACGTGATTGGTTA | |
| DSS3_cis2_46F | TTGGTCGATGGCGTGAAACA | |
| DSS3_cis2_129R | GGCAAGATGTTCGTTGCCAA | |
| DSS3_cis52_66F | ATATTGCGCCGTCATCACCT | |
| DSS3_cis52_166R | AGAACTTCTTCCTGCCGGTG | |
| DSS3_cis64_42F | TGGACATCAGCACGAACCTC | |
| DSS3_cis64_159R | ACGCCTTCGAAATTGGCAAC | |
| DSS3_cis67_279F | CAGAAGGCTCCCACAGTGAG | |
| DSS3_cis67_402R | CCGCTCAGTCTGTTGGAGAG | |
| DSS3_cis8_14F | TGGCGATGTATGGGATCAGC | |
| DSS3_cis8_106R | ATCCGCAGATCTTTTGGGCA | |
| DSS3_cis90_44F | TGCATCGGATCGAAGTAGCC | |
| DSS3_cis90_137R | CCGAGGCGGTCTATCTGTTC | |
| DSS3_trans43_218F | ACTGTGGTTGGGAAAACGGT | |
| DSS3_trans43_301R | GTGACGGCAACACAAGGAAC | |
| gyrA_365F | GCAATTTCGGCTCGATGGAC | |
| gyrA_486R | GTCCTGGAAGTCGACGGTTT | |
| rpoC_2059F | TGGTCCAAGTGTAACGACCG | |
| rpoC_2170 R | GGGCCATCATGTAGACCGAG | |
